# Metal organic framework-loaded polyethersulfone/polyacrylonitrile photocatalytic nanofibrous membranes under visible light irradiation for the removal of Cr(vi) and phenol from water

**DOI:** 10.1039/d3ra00959a

**Published:** 2023-04-25

**Authors:** Shahnaz Koushkbaghi, Hamta Arjmand Kermani, Sana Jamshidifard, Hamed Faramarzi, Mina Khosravi, Parvaneh Ghaderi-shekhi Abadi, Fariborz Sharifian Jazi, Mohammad Irani

**Affiliations:** a Science and Research Branch, Islamic Azad University Yazd Iran; b Faculty of Chemical Engineering, Iran University of Science & Technology Tehran Iran; c Chemical Engineering Departments, Razi University Kermanshah Iran; d Department of Environmental Engineering, Graduate Faculty of Environment, University of Tehran Tehran Iran; e Environmental Health Engineering Research Center, Alborz University of Medical Sciences Karaj Iran; f School of Science and Technology, The University of Georgia Tbilisi Georgia; g Department of Pharmaceutics, Faculty of Pharmacy, Alborz University of Medical Sciences Karaj Iran Irani_mo@ut.ac.ir

## Abstract

In this work, various amounts of the UiO-66-NH_2_ and UiO-66-NH_2_/TiO_2_ MOFs have been loaded into polyacrylonitrile (PAN) nanofibers supported on polyethersulfone (PES). The visible light irradiation was used to investigate the influence of pH (2–10), initial concentration (10–500 mg L^−1^), and time (5–240 min) on the removal efficiency of phenol and Cr(vi) in the presence of MOFs. The reaction time: 120 min, catalyst dosage: 0.5 g L^−1^, pH: 2 for Cr(vi) ions and pH: 3 for phenol molecules were optimum to degrade phenol and to reduce Cr(vi) ions. The characterization of the produced samples was performed using X-ray diffraction, ultraviolet-visible diffuse reflectance spectroscopy, scanning electron microscopy, and Brunauer–Emmett–Teller analysis. The capability of synthesized photocatalytic membranes was investigated for the removal of phenol and Cr(vi) ions from water. The water flux, Cr(vi) and phenol solutions fluxes and their rejection percentages were evaluated under pressure of 2 bar in the presence of visible light irradiation and in the dark. The best performance of the synthesized nanofibers was obtained for UiO-66-NH_2_/TiO_2_ MOF 5 wt% loaded-PES/PAN nanofibrous membranes at temperature of 25 °C and pH of 3. Results demonstrated the high capability of MOFs-loaded nanofibrous membranes for the removal of various contaminants such as Cr(vi) ions and phenol molecules from water.

## Abstract 2

Metal organic framework-loaded photocatalytic membrane to remove phenol and Cr(vi).

## Graphical abstract

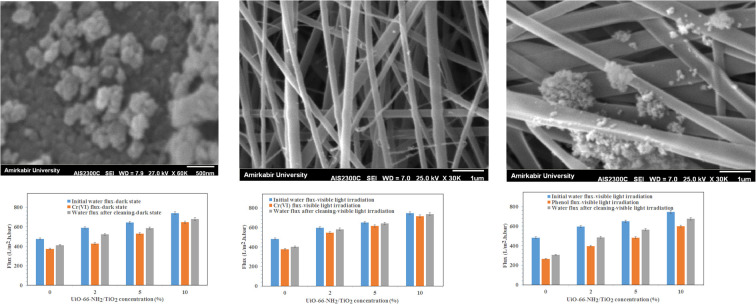

## 1. Introduction

The rapid development of industry and shortage of water resources caused the development of novel alternative faster methods for the rapid removal of contaminants from water.^1^ Various technologies including advanced oxidation processes (AOPs), membrane separation, coagulation, adsorption, and ion exchange have been used to remove toxic matters from water.^2^ Recently, hybrid methods such as adsorption/photocatalysis,^3–5^ coagulation/adsorption^6,7^ and photocatalysis/membrane^8–11^ have been developed to increase the removal efficiency of effluents and accelerate their treatment compared with simple treatment techniques. The photocatalysis/membrane technique is a physical separation/chemical oxidation combined method for the reduction of membrane fouling and increasing the removal efficiency of membranes.^10^

Metal organic frameworks (MOFs) as novel photocatalysts have been utilized for degrading organic effluents and reducing metal ions, due to their adjustable pores, high surface area, and high photocatalytic activity through the charge transfer between organic ligand–metal cluster under visible light irradiation.^12–16^ MOFs used for photo-degradation of toxic matters from aquatic systems include various types of UiO, MIL, and ZIF.^17^ However, the use of pure MOFs due to difficult recycling after the photocatalysis process is limited.^18–20^ The MOFs loaded membranes and development of photocatalytic membranes is an effective method for (I) uniform disposition of MOFs on the support, (II) use of MOFs in large-scale experiments, (III) prevention of their agglomeration during the photocatalysis process, and (IV) easier recycling after removal of effluents.^21^ For instance, Du *et al.*^22^ investigated the performance of UiO-66-NH_2_ membrane supported on α-Al_2_O_3_ under sunlight irradiation for reduction of Cr(vi) ions. Liu *et al.*^23^ incorporated the Ni@UiO-66 MOFs into the polyethersulfone (PES) membrane under UV irradiation for the water treatment. Ahmadi *et al.*^24^ immobilized 0.2 wt% NH_2_-MIL125(Ti) MOF on the polysulfone membrane for photodegradation of methylene blue under UV irradiation. They also suspended the MOF nanoparticles in the reactor. The methylene blue removal efficiency and flux recovery ratio were 97% and 88%, respectively. Sun *et al.*^25^ incorporated the poly(sulfobetaine methacrylate)/UiO-66 composite into the polysulfone ultrafiltration membrane. The water flux of the MOF-based composite-incorporated polysulfone was higher than that of the polysulfone membrane (about 2.5 times). Salehian *et al.*^26^ investigated the removal efficiency of natural organic matter using a TiO_2_@MIL-88A (Fe)-loaded polyacrylonitrile photocatalytic membrane. The humic acid removal efficiency and flux recovery ratio of the membrane were 92.4% and 99.5%, respectively. The nanofibers prepared by electrospinning are good candidates for incorporating MOFs.^27,28^ In recent years, the nanofibrous mats have been extensively utilized as a membrane in ultrafiltration, microfiltration, nanofiltration and forward osmosis membrane processes.^29–32^ However, the use of nanofibers in the continuous wastewater treatments such as membrane processes due to their low mechanical stability is limited. For instance, Khalil *et al.*^33^ investigated the potential of PAN/SiO_2_–TiO_2_–NH_2_ composite nanofibers for degradation of acid red 27 and malachite green under visible light. The rapid degradation of acid red 27 and malachite green using nanofibers was occurred during 9 and 25 min, respectively. In another study, the performance of a SiO_2_–TiO_2_-loaded polyaniline nanofiber membrane was studied to degrade the methyl orange.^34^ The prepared PAN/Ag–TiO_2_ nanofiber membrane indicated the high photocatalytic activity for the complete removal of methylene blue within 1 h.^35^ Pu *et al.*^36^ investigated the degradation of ciprofloxacin using a PAN/ZIF-65 MOFs nanofiber membrane. However, there is no study on the removal of phenol and Cr(vi) using polyethersulfone (PES)/PAN/UiO-66-NH_2_/TiO_2_ MOFs nanofiber membranes. In this work, the synthesized UiO-66-NH_2_/TiO_2_ MOFs were first loaded into the polyacrylonitrile (PAN) solution. The PAN/MOFs have been electrospun on the PES nanofibrous support to prepare the PES/PAN/MOFs photocatalytic nanofibrous membranes. The capability of synthesized photocatalytic membrane was investigated for the removal of phenol and Cr(vi) ions from water under visible light irradiation.

## 2. Experimental

### 2.1 Materials

Polyacrylonitrile (PAN, Mw = 150 kDa, Sigma-Aldrich, USA), polyether sulfone (PES, Mw = 58 kDa, Ultrason E6020P), 2-aminoterephthalic acid (purity ≥ 99.9%, Sigma-Aldrich, USA, BDC-NH_2_), zirconium chloride (purity 99%, Sigma-Aldrich, USA, ZrCl_4_), *N*,*N*-dimethylformamide (Merck, Germany, DMF), hydrochloric acid (HCL, 37%, Merck, Germany), and Titanium tetrabutoxide (C_16_H_36_O_4_Ti, purity 97%, Sigma-Aldrich, USA) were used for the preparation of nanofibrous membranes.

### 2.2 Synthesis of MOFs

UiO-66-NH_2_ and TiO_2_ nanoparticles were synthesized using hydrothermal and sol–gel methods as described previously.^19,37^ To prepare UiO-66-NH_2_/TiO_2_ composites, first 50 mg TiO_2_ nanoparticles were dispersed in ethanol. Then, 50 mg UiO-66-NH_2_ was dispersed in solution under sonication for 30 min. After that, the synthesized hybrid was filtered and washed three times with water and ethanol. Finally, the produced solid was dried at 100 °C overnight.

### 2.3 Fabrication of PES/PAN/MOFs membrane

The PES nanofibrous support was prepared by electrospinning method by dissolving 2 g PES in 8 mL DMF and its electrospinning under feeding rate of 1 mL h^−1^, voltage of 20 kV, and distance of 15 cm. PAN solution was prepared by its dissolving in DMF at 60 °C within 4 h. To prepare the PAN/MOFs and PAN/MOFs/TiO_2_ solutions, different amounts of MOFs and MOFs/TiO_2_ (2, 5 and 10 wt% by weight of PAN) were dispersed in DMF. Then, PAN was added under stirring overnight. First, the prepared PAN/MOFs and PAN/MOFs/TiO_2_ solutions were sonicated for 30 min, and then were electrospun on the PES support.

### 2.4 Photocatalytic experiments using MOFs

In the photocatalytic removal of phenol and Cr(vi) using MOFs, the impact of initial concentrations of phenol and Cr(vi) (10–500 mg L^−1^), pH (2–10), and contact time (5–240 min) on their removal using MOFs was investigated under Xenon irradiation (300 W, *λ* ≥ 420 nm, Beijing Aulight Co., Ltd).

### 2.5 Photocatalytic membrane experiments

**Scheme 1:**
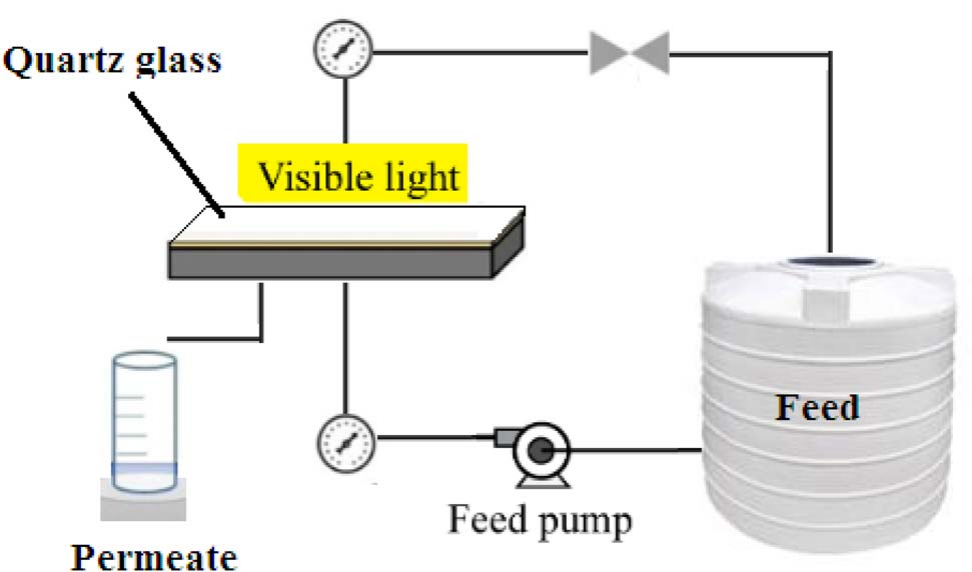
Experimental set-up of photocatalytic membrane process.

The performance of the PES/PAN/MOFs nanofibrous membranes was examined in a cross-flow photocatalytic membrane reactor under visible light (Xenon arc lamp), operating pressure of 2 bar, effective surface area of 35 cm^2^, and temperature of 25 °C. The filtration was carried out for 120 min with an initial feed concentration of 10 mg L^−1^. The membranes were regenerated using 0.1 M HCl solution (200 mL) for 2 h.^19^ The experimental set-up of the photocatalytic membrane process is illustrated in Scheme 1.

### 2.6 Characterization tests

The morphology of membranes was detected by employing a scanning electron microscopy (SEM) using, JEOL JSM-6380 microscope. An Image J software (Image-Proplus, Media Cybrernetics) was used to determine the particle size and the size distribution of particles and nanofibers. A diffuse reflectance spectrum (DRS) of MOFs was recorded using UV-2550 (Shimadzu, Japan) UV-vis spectrophotometer. The crystallinity and surface area of synthesized MOFS were determined using X-ray diffractometer type Philips PW 1730 (Japan) and Brunauer–Emmett–Teller (BET) analysis. The contact angle of membranes was investigated using a contact angle meter (CA-VP, Kyowa Interface Science Co., Ltd, Japan). The pore radius (*r*_m_) of nanofibrous membranes is calculated as follows:1
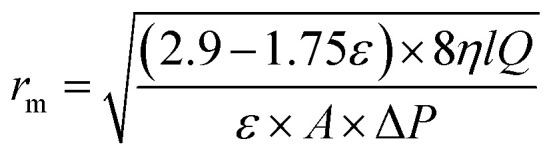
where *η* is the water viscosity (8.9 × 10^−4^ Pa s), *Q* is the volume of the permeate pure water per unit time (m^3^ S^−1^), Δ*P* is the operating pressure (0.2 MPa), *A* is the membrane effective area (m^2^), *l* is the thickness of the membrane (*m*) and *ε* is the porosity of the membrane which is defined as follows:2
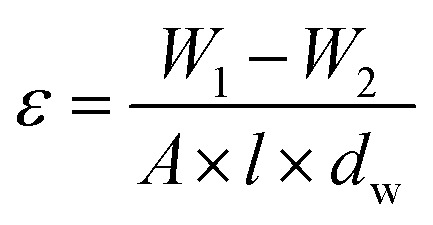
where *W*_1_ is the weight of the wet membrane, *W*_2_ is the weight of the dry membrane and *d*_w_ is the water density (0.998 g cm^−3^).

## 3. Results and discussion

### 3.1 Characterization of MOFs

**Fig. 1:**
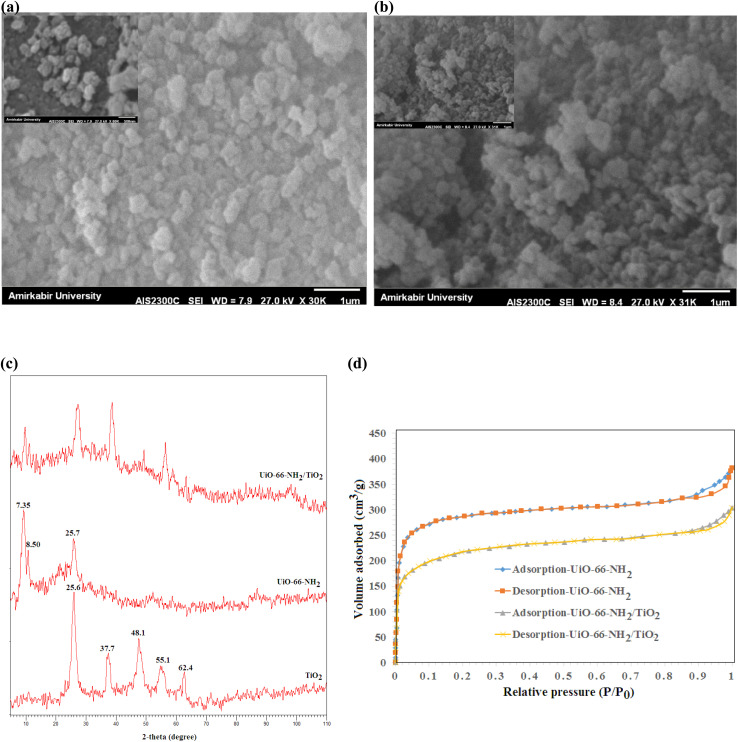
SEM images of (a) UiO-66-NH_2_ (b) UiO-66-NH_2_/TiO_2_ composite, (c) XRD patterns of UiO-66-NH_2_ and UiO-66-NH_2_/TiO_2_ composites and (d) N_2_ adsorption/desorption cycles of UiO-66-NH_2_ and UiO-66-NH_2_/TiO_2_ composites.

The SEM images of UiO-66-NH_2_ and UiO-66-NH_2_/TiO_2_ composites are illustrated in Fig. 1a and b. The particle sizes ranging from 150–250 nm with an average size of 185 ± 45 nm were obtained for UiO-66-NH_2_ MOFs. By blending TiO_2_ nanoparticles and UiO-66-NH_2_, the particle sizes ranging from 50–200 nm with an average size of 95 nm have been produced for UiO-66-NH_2_/TiO_2_ composite. The XRD patterns of synthesized MOFs are illustrated in Fig. 1c. For UiO-66-NH_2_ MOF nanoparticles, the characteristic peaks at 7.35°, 8.50° and 25.7° indicated the successful synthesis of UiO-66-NH_2_.^19^ For pure TiO_2_ nanoparticles, the detected peaks at 25.6°, 37.7°, 48.1°, 55.1° and 62.4° corresponding to the (1 0 1), (0 0 4), (2 0 0), (2 1 1) and (2 0 4) lattice planes demonstrated the anatase phase of TiO_2_ nanoparticles.^37^ The main peaks of UiO-66-NH_2_ and TiO_2_ nanoparticles were matched in the XRD pattern of UiO-66-NH_2_/TiO_2_ composite. The N_2_ adsorption/desorption cycles in the structure of UiO-66-NH_2_ and UiO-66-NH_2_/TiO_2_ MOFs are illustrated in Fig. 1d. The surface area of UiO-66-NH_2_ and UiO-66-NH_2_/TiO_2_ MOFs were found to be 825.2 and 410.1 m^2^ g^−1^, respectively. By blending UiO-66-NH_2_ and TiO_2_, some TiO_2_ nanoparticles were aggregated on the UiO-66-NH_2_ surface and decreased the BET surface area and pore volume of UiO-66-NH_2_. Furthermore, the lower surface area of TiO_2_ nanoparticles compared to UiO-66-NH_2_ MOFs resulted in the lower surface area of UiO-66-NH_2_/TiO_2_ in comparison to pure UiO-66-NH_2._

### 3.2 Characterization of nanofibrous membranes

**Fig. 2:**
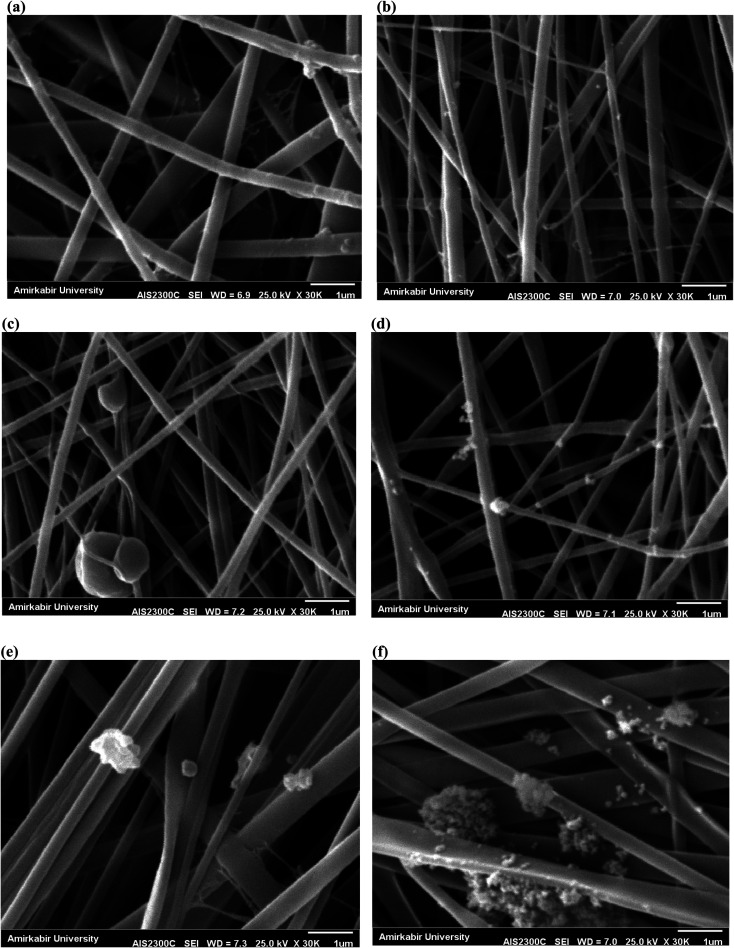
SEM images of (a) PES, (b) PAN, (c) PAN/UiO-66-NH_2_ 5%, (d) PAN/UiO-66-NH_2_/TiO_2_ 2%, (e) PAN/UiO-66-NH_2_/TiO_2_ 5%, and (f) PAN/UiO-66-NH_2_/TiO_2_ 10%.

The SEM images of the surface of PES nanofibrous support and PAN nanofibrous membranes with different content of MOFs (0, 2, 5 and 10 wt%) are presented in Fig. 2. The homogeneous nanofibers with average diameters of 360 ± 60 nm and 250 ± 50 nm were obtained for pure PES (Fig. 2a) and PAN (Fig. 2b) nanofibers, respectively. By loading 5 wt% UiO-66-NH_2_, some MOFs were observed on the nanofibers surface and the average diameter of nanofibers was increased to 330 ± 120 nm (Fig. 2c). The similar morphology with an average diameter of 315 ± 100 nm was obtained for 5 wt% UiO-66-NH_2_/TiO_2_-loaded PAN nanofibers (Fig. 2e). By loading 2 wt% UiO-66-NH_2_/TiO_2_ MOFs, the thinner fibers with average diameter of 280 ± 60 nm have been prepared and the most of MOFs nanoparticles without aggregation have been successfully incorporated into the nanofibers (Fig. 2d). By loading 2 wt% UiO-66-NH_2_/TiO_2_ MOFs into the PAN nanofibers, the viscosity of electrospinning solution was increased which resulted in gradual increase in the fiber diameter of PAN/UiO-66-NH_2_/TiO_2_ 2 wt% (280 ± 60 nm) compared to pure PAN nanofibers (250 ± 50 nm). By increasing the concentration of UiO-66-NH_2_/TiO_2_ in the PAN solution, the aggregation of UiO-66-NH_2_/TiO_2_ nanoparticles in the solution and non-homogenous dispersion of nanoparticles resulted in the formation of UiO-66-NH_2_/TiO_2_ nanoparticles on the surface of the nanofibers. By loading 10 wt% UiO-66-NH_2_/TiO_2_ MOFs, most of MOFs were aggregated on the nanofibers surface (Fig. 2f). The electrospinning of PES/PAN/UiO-66-NH_2_/TiO_2_ MOFs with higher UiO-66-NH_2_/TiO_2_ concentrations than 10%, due to the high viscosity of solution and aggregation of nanoparticles in the solution before the electrospinning, was impossible. The other structural parameters of synthesized nanofibrous membranes is listed in Table 1. The water contact angle of pure PES/PAN, PES/PAN/UiO-66-NH_2_/TiO_2_ 2%, PES/PAN/UiO-66-NH_2_/TiO_2_ 5% and PES/PAN/UiO-66-NH_2_/TiO_2_ 10% nanofibrous membranes were found to be 77.3 ± 1.2°, 65.6 ± 1.4°, 49.8 ± 1.3°, and 38.8 ± 1.2°, respectively. The average thickness of nanofibrous membranes was about 75 ± 5 μm. The average pore size and porosity of pure PES/PAN nanofibers were 2.98 μm, and 72.3%. By loading UiO-66-NH_2_/TiO_2_ up to 5% into the nanofibrous membrane, the porosity and pore size of nanofibers was gradually increased and a further increase in the UiO-66-NH_2_/TiO_2_ content (10 wt%) resulted in decreasing the porosity and pore size of nanofibrous membranes. The increase in the porosity of nanofibers by loading of UiO-66-NH_2_/TiO_2_ could be attributed to the higher porosity of UiO-66-NH_2_/TiO_2_ in the nanofibers. The decrease in the porosity and pore size of nanofibers containing 10 wt% UiO-66-NH_2_/TiO_2_ could be attributed to the nanoparticles aggregation and there are not enough free voids to equilaterally distribute the nanoparticles into the nanofibers, as confirmed by SEM image.

**Table 1:** Structural parameters of the fabricated nanofibrous membranes

Membrane	Water contact angle (°)	Pore size (μm)	Porosity (%)
PES/PAN	77.3 ± 1.2	2.98 ± 0.15	72.3 ± 1.3
PES/PAN/UiO-66-NH_2_/TiO_2_ 2%	65.6 ± 1.4	3.85 ± 0.13	76.7 ± 0.9
PES/PAN/UiO-66-NH_2_/TiO_2_ 5%	49.8 ± 1.3	5.25 ± 0.21	81.3 ± 1.1
PES/PAN/UiO-66-NH_2_/TiO_2_ 10%	38.8 ± 1.2	1.96 ± 0.10	78.5 ± 1.9

### 3.3 Photocatalytic removal of Cr(vi) and phenol in a batch system

**Fig. 3:**
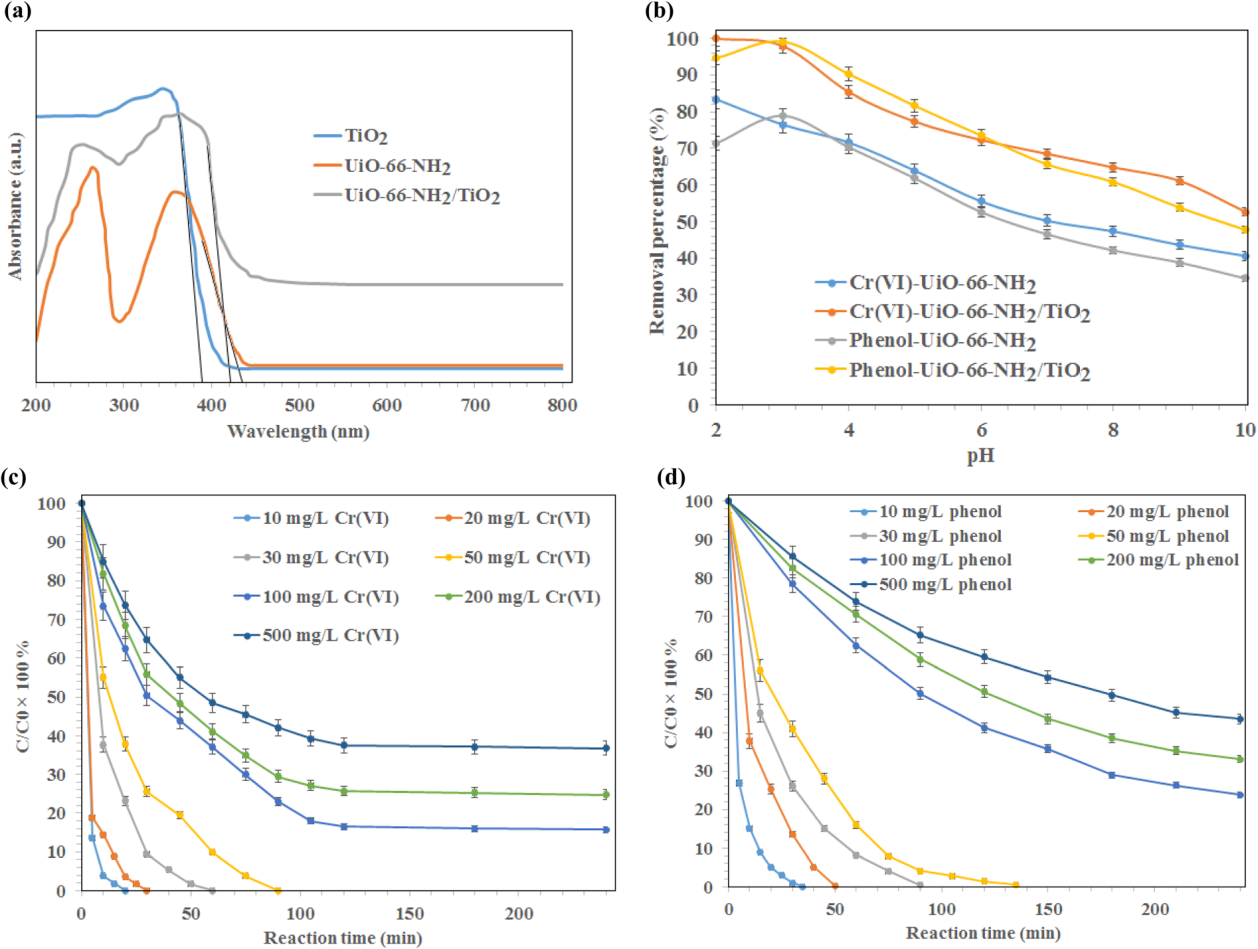
(a) UV-DRS spectra of synthesized TiO_2_, UiO-66-NH_2_, and UiO-66-NH_2_/TiO_2_ MOFs, (b) effect of pH on the photo-degradation of Cr(vi) and phenol using UiO-66-NH_2_, and UiO-66-NH_2_/TiO_2_ MOFs under visible light irradiation, and the effect of reaction time on the removal of (c) Cr(vi) and (d) phenol using UiO-66-NH_2_/TiO_2_.

The UV diffuse reflectance spectra (DRS) of synthesized MOFs are illustrated in Fig. 3. As shown, the absorption edge of TiO_2_, UiO-66-NH_2_, and UiO-66-NH_2_/TiO_2_ MOFs were found to be 388.6 nm, 435.1 nm, and 421.8 nm respectively, indicating that UiO-66-NH_2_, and UiO-66-NH_2_/TiO_2_ MOFs could be activated under visible light irradiation. The band-gap energy of UiO-66-NH_2_, and UiO-66-NH_2_/TiO_2_ MOFs was estimated to be 2.85 eV and 2.94 eV, respectively.

The effect of pH on the photo-degradation of phenol and Cr(vi) using MOFs under visible light, catalyst dosage of 0.5 g L^−1^, initial concentration of 10 mg L^−1^, reaction time of 240 min, temperature of 25 °C, and pH values ranging from 2–10 is illustrated in Fig. 3b. As shown, the maximum removal of Cr(vi) using UiO-66-NH_2_, and UiO-66-NH_2_/TiO_2_ MOFs was occurred at pH 2. At lower pH values, the better reduction of Cr_2_O_7_^2−^ ions was occurred, due to the better electrostatic attraction of Cr(vi) anions and synthesized MOFs. After that, the removal of Cr(vi) ions was occurred by irradiation of visible light on the MOFs surface *via* the photogenerated-electron–hole pairs (eqn (3) and (4)). At higher pH values, the precipitation of chromium anions in the form of Cr(OH)_3_ might cove the active sites of synthesized photocatalysts and reduced their photocatalytic efficiency (eqn (5)).

3 Cr_2_O^2−^_7_ + 14H^+^ + 6e^−^ → 2Cr^3+^ + 7H_2_O

4 4h^+^ + 2H_2_O → O_2_ + 4H^+^

5 CrO^2−^_4_ + 4H_2_O + 3e^−^ → Cr(OH)_3_ + 5OH^−^

The optimum pH for the removal of phenol using synthesized photocatalysts was occurred at pH 3. As shown, the complete degradation of phenol was obtained using UiO-66-NH_2_/TiO_2_ MOFs at pH 3 after 120 min. The maximum phenol removal percentages in the presence UiO-66-NH_2_, and UiO-66-NH_2_/TiO_2_ MOFs were 81.3% and 99.5%, respectively. Therefore, the pH values of 2 and 3 were selected for further experiments.

The effect of reaction time on the removal of Cr(vi) ions and phenol at various concentrations (10–500 mg L^−1^) using UiO-66-NH_2_/TiO_2_ MOFs is illustrated in Fig. 3c and d. As shown, the complete reduction of chromium ions by UiO-66-NH_2_/TiO_2_ MOFs for initial concentrations of 10, 20, 30, and 50 mg L^−1^ was occurred after 20, 30, 60 and 90 min, respectively. The maximum Cr(vi) removal percentages for initial concentrations of 100, 200 and 500 mg L^−1^ Cr(vi) ions were found to be 83.5% ± 1.5%, 74.3% ± 1.7% and 62.5% ± 2.1%, respectively, after 120 min. The phenol removal percentage higher than 99% was obtained using UiO-66-NH_2_/TiO_2_ MOFs under initial concentrations of 10, 20, 30, and 50 mg L^−1^ after 35 min, 50 min, 90 min and 135 min, respectively (Fig. 3d). The maximum phenol degradation was found to be 76.2% ± 1.3%, 66.9% ± 1.8% and 56.5% ± 1.9%, respectively, after 240 min for initial concentrations of 100, 200 and 500 mg L^−1^ phenol. The higher removal percentages of Cr(vi) and phenol using UiO-66-NH_2_/TiO_2_ MOFs than the UiO-66-NH_2_ could be attributed to the higher photocatalytic activity of UiO-66-NH_2_/TiO_2_ composite. Although, the pure UiO-66-NH_2_ MOFs exhibited the higher specific surface area and lower band-gap energy compared to UiO-66-NH_2_/TiO_2_ composite, the contact interfaces between TiO_2_ and UiO-66 promoted the separation/migration efficiency of photogenerated electron/hole pairs during photocatalytic reaction and resulted in increasing the photocatalytic activity of UiO-66-NH_2_ for the removal of Cr(vi) and phenol from water.^38^

### 3.4 Photocatalytic membranes

**Fig. 4:**
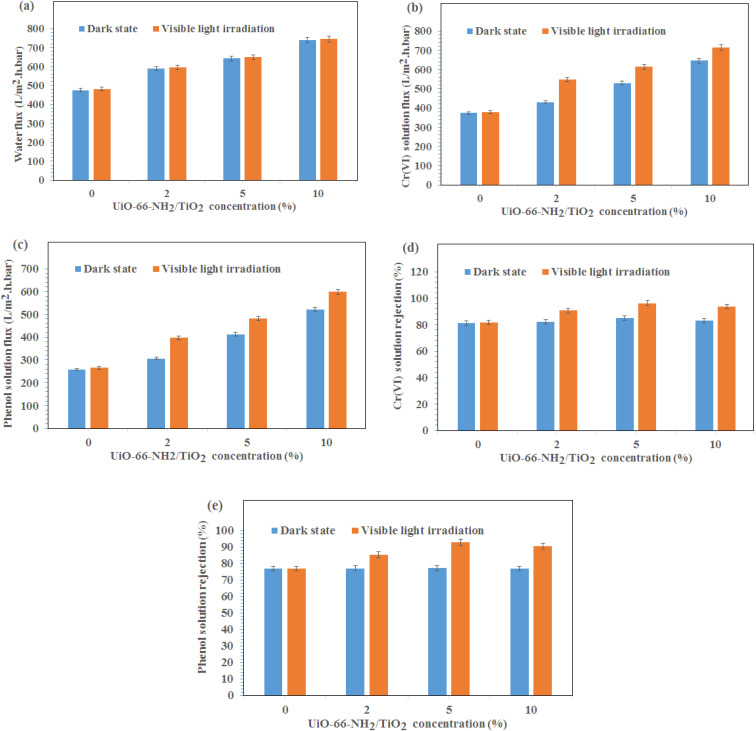
(a) Water permeation, (b) Cr(vi) solution flux, (c) phenol solution flux, (d) Cr(vi) rejection, (e) phenol rejection at the pressure of 2 bar under visible light irradiation and dark state.

The water permeation, Cr(vi) solution flux and phenol solution flux were evaluated at the pressure of 2 bar under visible light irradiation and without light irradiation (Fig. 4). As shown in Fig. 4a, the permeability of the PES/PAN nanofibrous membrane was increased by incorporating UiO-66-NH_2_/TiO_2_ MOFs into the PES/PAN membrane. The water permeability of PES/PAN, PES/PAN/UiO-66-NH_2_/TiO_2_ 2%, PES/PAN/UiO-66-NH_2_/TiO_2_ 5% and PES/PAN/UiO-66-NH_2_/TiO_2_ 10% nanofibrous membranes was found to be 475.2 L m^−2^ h^−1^ bar^−1^, 589.4 L m^−2^ h^−1^ bar^−1^, 643.6 L m^−2^ h^−1^ bar^−1^, and 739.3 L m^−2^ h^−1^ bar^−1^, respectively. By increasing the concentration of UiO-66-NH_2_/TiO_2_, the hydrophilicity of membrane was increased which resulted in increasing the water permeability. The water contact angle of pure PES/PAN, PES/PAN/UiO-66-NH_2_/TiO_2_ 2%, PES/PAN/UiO-66-NH_2_/TiO_2_ 5% and PES/PAN/UiO-66-NH_2_/TiO_2_ 10% nanofibrous membranes were found to be 77.3 ± 1.2°, 65.6 ± 1.4°, 49.8 ± 1.3°, and 38.8 ± 1.2°, respectively. The enrichment of the surface of membranes with –NH_2_ and Ti–O groups, resulted in decreasing of the water contact angle and increasing the hydrophilicity of membranes by increasing UiO-66-NH_2_/TiO_2_ concentration in the PES/PAN membrane. Furthermore, the loading of UiO-66-NH_2_/TiO_2_ with high porosity into the membrane may be increased the membrane porosity and enhanced the water permeability of PES/PAN nanofibrous membrane. The light irradiation did not impact on the water permeability. This behavior indicated no significant reaction between the hydroxyl radicals and polymer chains. Therefore, the intrinsic resistance of the membrane exhibited a critical role on the water permeability. The blocking of some pores of nanofibrous membranes with phenol and Cr(vi) resulted in a gradual decrease of phenol and Cr(vi) solutions compared with the water permeability of PES/PAN/UiO-66-NH_2_/TiO_2_ MOFs nanofibrous membranes (Fig. 4b and c). The Cr(vi) and phenol solutions fluxes have been increased in the presence of visible light irradiation. The photocatalytic degradation of Cr(vi) ions and phenol molecules that blocked the nanofibers pores, resulted in improving the Cr(vi) and phenol solutions permeability under visible light.

The gradual enhancement of Cr(vi) rejection by increasing the concentration of UiO-66-NH_2_/TiO_2_ was due to the increasing the hydrophilicity under dark state (Fig. 4d). However, the rejection of phenol did not significantly change by loading of UiO-66-NH_2_/TiO_2_ (Fig. 4e). The photodegradation of phenol and Cr(vi) ions by hydroxyl radicals resulted in increasing removal efficiencies of phenol and Cr(vi) under visible light (Fig. 4d and e). The removal efficiencies of phenol and Cr(vi) using PES/PAN/UiO-66-NH_2_/TiO_2_ 5% were 84.9 and 77.3% under dark state. Whereas, the maximum removal efficiencies of phenol and Cr(vi) were 92.7 and 96.3% in the presence of PES/PAN/UiO-66-NH_2_/TiO_2_ 5% nanofibrous membrane under visible light. The gradual decrease in the phenol and Cr(vi) rejection percentages by increasing UiO-66-NH_2_/TiO_2_ concentration up to 10 wt% may be attributed to the increase in the membrane porosity and pore radius. Similar trend is reported by Ahmadipouya *et al.*.^39^ They found that the mixed-matrix membrane containing 9 wt% UiO-66 was optimum for the removal of dyes and further loading of UiO-66 MOFs (12 wt%) resulted in decreasing the rejection percentages of dyes.

**Fig. 5:**
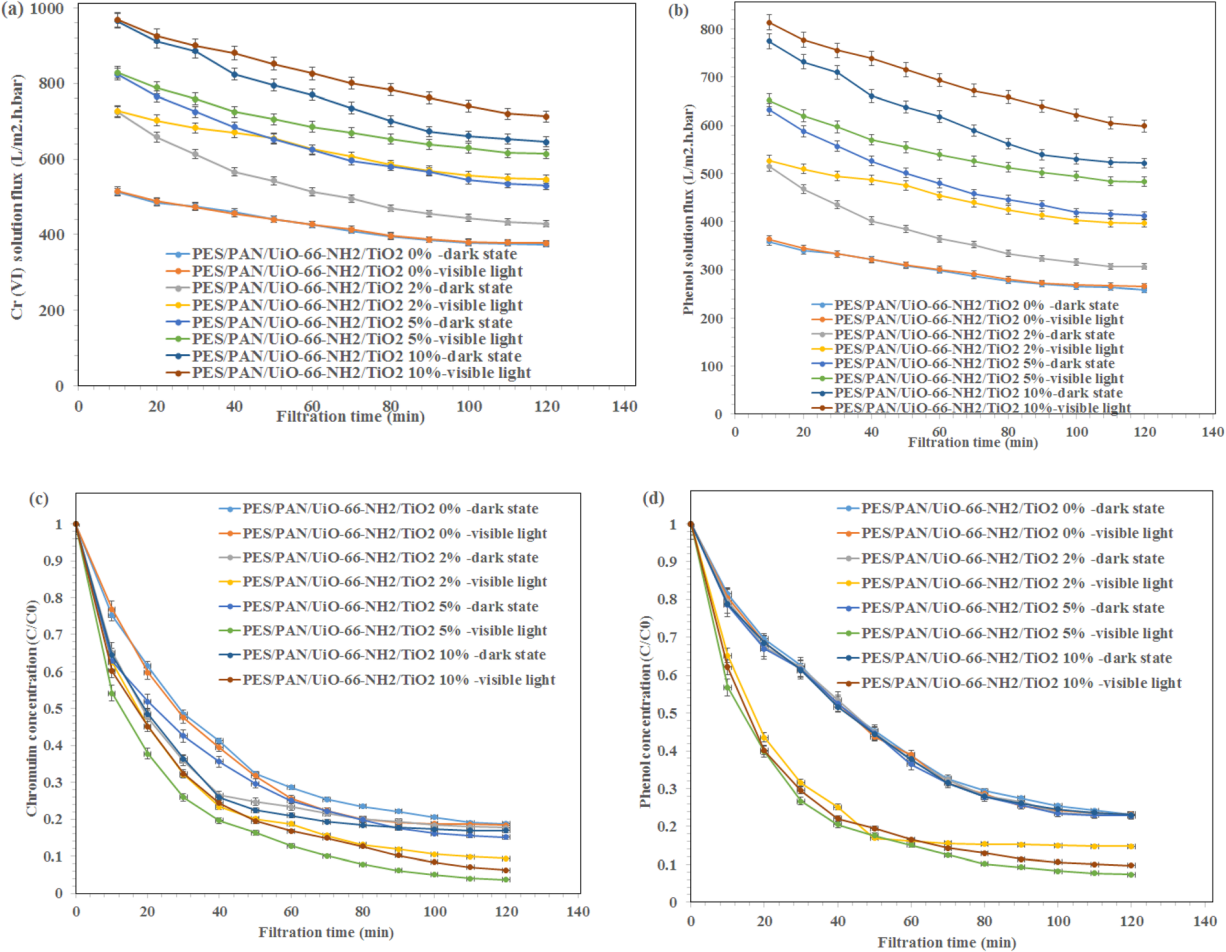
(a) Cr(vi) and (b) phenol solutions fluxes during 120 min and (c) Cr(vi) and (d) phenol rejection percentages during 120 min in the presence visible light irradiation and dark state.

The phenol solution flux, Cr(vi) solution flux, phenol rejection and Cr(vi) rejection during 120 min in the presence visible light irradiation and without light irradiation are presented in Fig. 5. The fluxes of phenol and Cr(vi) have decreased from 824.3 L m^−2^ h^−1^ bar^−1^ to 529.6 and 633.1 to 412.3 L m^−2^ h^−1^ bar^−1^ for phenol and Cr(vi) ions solutions using PES/PAN/UiO-66-NH_2_/TiO_2_ 5% nanofibrous membrane in the dark state. The higher hydrophilicity of nanofibrous membrane containing 5 wt% UiO-66-NH_2_/TiO_2_ compared to the hydrophilicity of composite membranes containing lower amounts of UiO-66-NH_2_/TiO_2_ resulted in its lower flux decline. At higher amounts of UiO-66-NH_2_/TiO_2_, the interaction between contaminants and membrane surface resulted in its garadual higher flux decline compared with 10 wt% UiO-66-NH_2_/TiO_2_ loaded- PES/PAN nanofibrous membrane. In the presence visible light, the flux decline has been improved and the minimum flux decline was found to be 26.0% and 25.8% for Cr(vi) and phenol solutions using PES/PAN/UiO-66-NH_2_/TiO_2_ 5% nanofibrous membrane. The hydroxyl radicals generated during photocatalytic reaction could degrade the phenol molecules and Cr(vi) ions and could prevent the flux decline.

The maximum Cr(vi) and phenol rejection percentages were 84.9 and 77.3% under dark state using PES/PAN/UiO-66-NH_2_/TiO_2_ 5% which were due to the adsorption of contaminants by the membrane and a further removal of Cr(vi) and phenol under visible light (phenol: 92.7% and Cr(vi) 96.3%) were due to the photocatalytic reduction of contaminants. Therefore, the prepared nanofibrous membranes could eliminate Cr(vi) and phenol from water through the adsorption, filtration, and photocatalytic reduction. For the phenol degradation, the removal efficiency did not significantly change by increasing UiO-66-NH_2_/TiO_2_ concentration under the dark state. However, the degradation ability of PES/PAN/UiO-66-NH_2_/TiO_2_ was enhanced by increasing UiO-66-NH_2_/TiO_2_ content up to 5%, which due to the enhanced photocatalytic capacity of PES/PAN nanofibers. Therefore, UiO-66-NH_2_/TiO_2_ as a photocatalysis composite could improve the performance of PES/PAN/UiO-66-NH_2_/TiO_2_ nanofibrous membrane to degrade the phenol molecules. For Cr(vi) reduction, the removal efficiency of PES/PAN/UiO-66-NH_2_/TiO_2_ was increased by loading UiO-66-NH_2_/TiO_2_ into the membrane up to 5% under both dark state and visible light irradiation. Therefore, the adsorption capacity, and photocatalytic reduction of membrane have been improved for reducing Cr(vi) ions from water. The obtained results indicated that the prepared photocatalytic membrane exhibited a better photocatalytic performance to eliminate Cr(vi) and phenol under visible light irradiation.

**Fig. 6:**
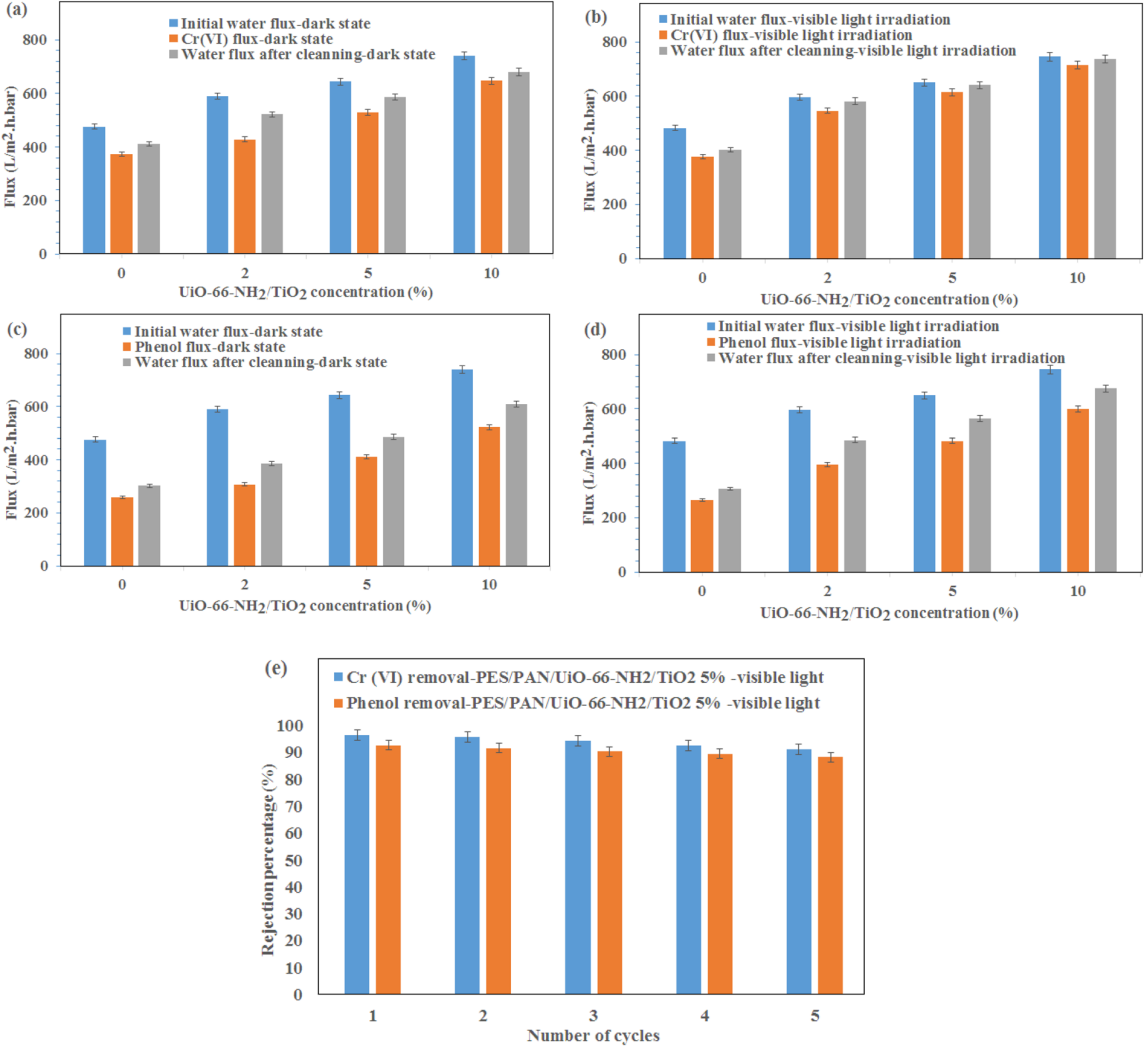
The change in the equilibrium fluxes of (a) water–Cr(vi) in the dark state, (b) water–Cr(vi) under visible light irradiation, (c) water–phenol in the dark state, (d) water–phenol under visible light irradiation after regeneration of nanofibrous membranes with 0.1 M HCl and (e) Cr(vi) and phenol removal using PES/PAN/UiO-66-NH_2_/TiO_2_ 5% nanofibrous membrane under visible light for five cycles.

The change in the equilibrium fluxes after regeneration of nanofibrous membranes with 0.1 M HCl are illustrated in Fig. 6. As shown, the flux recovery of MOFs-loaded membranes under visible light irradiation was higher than that of the dark state, due to the photocatalytic reactions inside the pores resulting in the enhanced dissolution of the membrane fouling in water, which in turn improved the water flux after cleaning under visible light irradiation.^40^ The equilibrium fluxes of composite nanofibous membrane containing 5 wt% UiO-66-NH_2_/TiO_2_ was maximum before and after rising with HCl under visible light irradiation. This behavior indicated the effect of metal organic framework as a porous material and the photocatalytic reaction on the improvement the performance of the metal organic framework-based nanofibrous membrane. However, more studies are needed for the reduction of fouling of membranes in the presence photocatalytic reactions.

To investigate the stability of prepared membranes, the Cr(vi) and phenol rejection were investigated for five cycles using PES/PAN/UiO-66-NH_2_/TiO_2_ 5% nanofibrous membrane under visible light irradiation (Fig. 6e). As shown, the removal efficiencies of Cr(vi) and phenol did not significantly change even after five cycles which demonstrated the stability of the membranes for industrial applications in the future.

## 4. Conclusion

In this work, the UiO-66-NH_2_/TiO_2_ MOFs were synthesized *via* the hydrothermal method. The various amounts of MOFs were incorporated into the PES/PAN nanofibers membranes to investigate the performance of nanofibrous membranes for the removal of Cr(vi) and phenol under visible light irradiation. The UiO-66-NH_2_ and UiO-66-NH_2_/TiO_2_ nanoparticles with average particle size of 185 ± 45 nm and 95 ± 25 nm were produced. The surface area of UiO-66-NH_2_ and UiO-66-NH_2_/TiO_2_ MOFs were found to be 825 and 410 m^2^ g^−1^. The average fiber diameter of PAN and PAN nanofibers containing 2,5 and 10 wt% UiO-66-NH_2_/TiO_2_ MOFs were found to be 250 ± 50 nm, 280 ± 60 nm, 315 ± 100 nm and 410 ± 140 nm, respectively. The maximum Cr(vi) removal percentages for initial concentrations of 100, 200 and 500 mg L^−1^ were found to be 83.5% ± 1.5%, 74.3% ± 1.7% and 62.5% ± 2.1%, respectively, after 120 min. The phenol removal percentage higher than 99% was obtained using UiO-66-NH_2_/TiO_2_ MOFs under initial concentrations of 10, 20, 30, and 50 mg L^−1^ after 35 min, 50 min, 90 min and 135 min, respectively. The water permeability of PES/PAN, PES/PAN/UiO-66-NH_2_/TiO_2_ 2%, PES/PAN/UiO-66-NH_2_/TiO_2_ 5% and PES/PAN/UiO-66-NH_2_/TiO_2_ 10% nanofibrous membranes was found to be 475.2 L m^−2^ h^−1^ bar^−1^, 589.4 L m^−2^ h^−1^ bar^−1^, 643.6 L m^−2^ h^−1^ bar^−1^, and 739.3 L m^−2^ h^−1^ bar^−1^, respectively. The removal efficiencies of phenol and Cr(vi) using PES/PAN/UiO-66-NH_2_/TiO_2_ 5% were 77.3 and 84.9% under dark state. Whereas, the maximum removal efficiencies of phenol and Cr(vi) were 92.7 and 96.3% in the presence of PES/PAN/UiO-66-NH_2_/TiO_2_ 5% nanofibrous membrane under visible light irradiation. The equilibrium fluxes of composite nanofibous membrane containing 5 wt% was maximum before and after rising with HCl under visible light irradiation. The obtained results demonstrated the high capability of MOFs in composite nanofibrous membrane for the removal of various contaminants from water during photocatalytic membrane process.

## Conflicts of interest

There are no conflicts to declare.

## Supplementary Material
